# News and Coming Events

**Published:** 1908-12-05

**Authors:** 


### NEWS AND COMING EVENTS.

MISS HOOD'S ball in aid of the West London Hospital will be held at the Grafton Galleries, on Wednesday, December 9, under the patronage of H.R.H. Princess Louise, Duchess of Argyll (President of the Ladies' Association of the Hospital). Particulars of the ball may be obtained of Miss Hood, 43 Green Street, Park Lane, W.

THE Duchess of Buccleuch, the President, took the chair at a general meeting of the Westminster Hospital Ladies' Association, held at the Hospital on November 25. Reports of the working of the Association were presented, and a satisfactory increase in the membership reported. It was announced that in aid of the funds of the Association a ball will be held at the Wharncliffe Rooms on February 17.

SIR ARTHUR CONAN DOYLE presided at Queen's Hall at the annual concert organised by Middlesex Hospital students in aid of the Cancer Wards Charity, and at the close of the concert, in the course of a brief speech, said that the charity had been established for one hundred years. The Middlesex Hospital is the only one in Great Britain which has a special institution of the kind attached to it. In the last year nearly three hundred cancer cases were treated at a cost of £4,500. The hospital has a special research laboratory, with a director and eight assistants, all endeavouring to prevent, as well as cure, this disease. He hoped that all present and all interested in the relief of suffering would subscribe to the charity.

AT the last interim Court of Governors of the Hospital for Consumption, Lord Cheylesmore in the chair, the committee of management reported that the scheme for voluntary notification, outlined in previous reports and at the Mansion House meeting, was now in operation, and was welcomed by the medical officers of health. The committee recorded that at the Washington International Congress on Tuberculosis the prize of $1,000 offered in competition against all countries for the best hospital exhibit of the treatment of advanced cases of consumption had been awarded to the Brompton Hospital, and that the Frimley Sanatorium had divided with the Whitehaven Sanatorium, Pennsylvania, the prize of $1,000 for the best exhibit of dealing with curable cases. The following legacies were announced :—Miss E. M. Hutchison, £2,500; Miss Ellen B. Wood, £100; Miss Anne Scott, £5,000.

THE annual meeting of the Royal Hospital for Incurables was held on November 27, Mr. H. J. Allcroft, the treasurer, presiding. The secretary submitted the fifty-fourth annual report. Last May, owing to the very large number of urgent cases waiting, thirty-five candidates were elected in place of the usual twenty-five. The result was that all the available beds were filled up, the number of in-patients being greater during the last six months than at any time during the history of the institution. There were now within the hospital 227 patients, and in addition there were 700 pensioners. Though the past year had been a successful one financially, the board felt that, with an annual expenditure of nearly £32,000, the income derived from investments—under £6,000—was still far short of what it ought to be. The board made an urgent appeal for new annual subscribers to take the place of those lost by death and other causes. The report was adopted. Subsequently thirty candidates were elected to the benefits of the institution.

MR. CHARLES SINGER, M.A., M.B., B.Ch.Oxon, has been appointed medical registrar, and Mr. E. H. Kettle, M.B., B.S.Lond., has been appointed assistant pathologist to the Cancer Hospital, Fulham Road, London.

THE quarterly report on the progress of the medical treatment of sleeping sickness in Uganda was issued on November 26. It deals with the work of the quarter which ended on February 29 last. In his introduction Dr. Hodges states that there is no doubt that the hopes expressed by Dr. Koch that atoxyl would prove a general and permanent cure for cases of sleeping sickness must now be abandoned, but that in most cases there is probably prolongation of life, and that the results from atoxyl and mercury may prove to be more lasting than those from atoxyl alone.

DR. WILLIAM ALFRED ELLISTON, a former President of the British Medical Association, died last week at Felixstowe. Born in 1840, Dr. Elliston, who was a son of Mr. William Elliston, M.R.C.S., was educated at Ipswich School and Guy's Hospital. He qualified M.R.C.S. and L.S.A. in 1862, and during that year he graduated M.D. at St. Andrews University. He soon enjoyed a large practice at Ipswich, and was in constant request as a consultant in the Eastern Counties. For upwards of thirty years he was surgeon to the Ipswich and East Suffolk Hospital, and on his retirement in 1900 was elected honorary consulting surgeon. He took a prominent part in the unsuccessful attempt to secure for members of the Royal College of Surgeons a share in its government. For many years Dr. Elliston was a member of the Council of the British Medical Association, and he became its president in 1900. He was also for a long period president of the Medical Defence Union. He was a magistrate for Suffolk and the first president of the Suffolk Liberal Unionist Association.

THE President (Major W. R. M. Thoyts) presided at a special Court of Governors of the Royal Berkshire Hospital, held at Reading on November 25, to consider a recommendation of the board of management to raise the necessary money for and to proceed at once with the proposed enlargement of the hospital. The scheme, which, it is estimated, will cost £20,000, provides for increased accommodation in the out-patients' departments, an extension of the waiting-hall, and the erection of a new dispensary and suites of rooms for physicians and surgeons. The president said that the accommodation had long been inadequate. The board had received an offer of £1,000 from an anonymous friend on condition that the work was begun before the next annual Court of Governors in February. The plans submitted were approved, and it was resolved to make an earnest effort to raise a sufficient sum to enable the work to be begun in time to secure the £1,000 offered. A sum not exceeding one-third of the estimated cost will be devoted towards the scheme from the invested funds of the hospital. Colonel J. E. Broadbent, C.B., hon. treasurer, announced gifts of £100 (conditional on the work being started by February) and £250. An analysis of the various accounts of the institution showed that, allowing for the estimated expenditure to the end of the financial year in January, there was a balance of £3,700 in hand. The invested funds amount to £52,000, and of this £11,000 could be devoted towards the extension scheme if the Governors so decided.

